# The thickness-dependent response of aerosol-jet-printed ultrathin high-aspect-ratio electrochemical microactuators†

**DOI:** 10.1039/d4sm00886c

**Published:** 2024-11-01

**Authors:** Ji Zhang, Jeremy J. Baumberg, Sohini Kar-Narayan

**Affiliations:** a Department of Materials Science & Metallurgy, University of Cambridge 27 Charles Babbage Road Cambridge CB3 0FS UK sk568@cam.ac.uk; b NanoPhotonics Centre, Cavendish Laboratory, University of Cambridge JJ Thomson Avenue Cambridge CB3 0HE UK jjb12@cam.ac.uk

## Abstract

Trilayer electrochemical actuators comprising an electrolyte layer sandwiched between two electrode layers have been shown to exhibit large deformations at low actuation voltages. Here we report the aerosol-jet printing (AJP) of high-aspect-ratio bending-type trilayer electrochemical microactuators comprised of Nafion as the electrolyte and poly(3,4-ethylenedioxythiophene)polystyrene sulfonate (PEDOT:PSS) as the electrode. We investigated how the thicknesses of the electrolyte and electrode layers affect the DC response of these actuators by fabricating high-aspect-ratio trilayer cantilevers with varied layer thicknesses (0.36 μm to 1.9 μm-thick electrodes, and 3.5 μm to 12 μm-thick electrolyte layers). We found that the transported charge and angular deflection are proportional to the applied voltage at steady state, and the charge-to-voltage ratio scales with the PEDOT:PSS thickness. The deflection-to-voltage ratio is found to be strongly affected by the Nafion electrolyte thickness, showing a decreasing trend, but is less affected by the PEDOT:PSS thickness in the range of dimensions fabricated. The timescales for deflection are found to be generally longer than the timescales for charge transfer and no clear trend is observed with respect to layer thicknesses. This work establishes an experimental protocol in geometry optimisation of printed electrochemical microactuators, verifies the applicability of a theoretical model, and lays the groundwork for designing and optimising more sophisticated printed electrochemical microactuation systems.

## Abstract 2

Using aerosol-jet printing, we fabricate and measure electrochemical microactuators with varying layer thicknesses, exploring how these variations influence their DC response.

## Graphical abstract

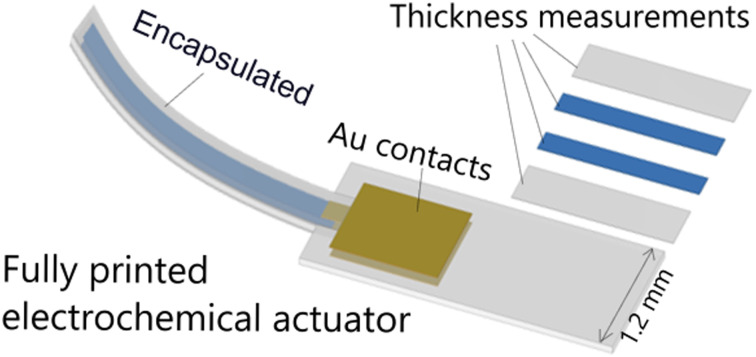

## Introduction

Trilayer electrochemical actuators have great potential in biomedical and biomimetic microrobotic applications due to their large deformation at low actuation voltages, ability to operate in both air and aquatic environments, biocompatibility, and suitability for miniaturisation.^1–4^ These actuators consist of a solid polymer electrolyte sandwiched between two electrodes and actuate as a result of applied electric field-driven ion migration. The actuation mechanism based on ion transfer and redistribution, however, leads to slow actuation speed and nonlinear response.^5,6^ Appropriate fabrication techniques, comprehensive modelling, and extensive experimental verification are essential for multi-objective optimisation of the actuator performance, a better understanding of the actuation behaviour, and accurate output prediction based on input.

The effect of the electrolyte and electrode thicknesses on the actuator performance has been studied on various trilayer electrochemical actuators. Lee *et al.* drop-cast Nafion electrolyte layers of different thicknesses (200 to 800 μm) in ionic polymer–metal composites (IPMCs) and observed that thicker IPMCs gave lower actuation displacements and higher blocking forces.^7^ He *et al.* reported the same trend, as well as an increase in elastic modulus with thicker cast Nafion, due to the prolonged annealing time and possibly increased cross-linking.^8^ Yilmaz *et al.* fabricated Au/Nafion/Au IPMCs by thermally evaporating Au of thicknesses from 10 nm to 80 nm.^9^ Since thicker Au resulted in less electrical resistance but higher rigidity, ∼45 nm thick Au gave faster and larger displacement as well as higher blocking force. Hui *et al.* fabricated IPMCs with 1.8 to 6 μm-thick Ag electrodes by electroless-plating 1 to 5 times, reporting maximum actuation displacement and force with 3 secondary electroless plating iterations for the same reasons.^10^ Oh *et al.* fabricated trilayer actuators with hot-pressed Nafion membranes and multi-walled carbon nanotube electrodes.^11^ They showed that the displacement was inversely proportional to the Nafion membrane thickness and the tip force increased with the Nafion thickness. At the same time, this work highlighted the effect of the hot-pressing technique used to fabricate the films on the crystallinity and mechanical properties of the membrane. Alici *et al.* developed a methodology for geometry optimisation of polypyrrole (PPy) trilayer actuators with a finite element model using the analogy between thermal strain and ion transport-induced strain.^12^ They electrodeposited PPy electrodes of different thicknesses on a 110 μm Pt-coated porous polyvinylidene fluoride (PVDF) electrolyte and found that the bending moment increased with the PPy thickness but decreased when the PPy was thicker than 60 μm. The simulation also showed that thicker PPy closer to the root of the actuator could increase the bending moment.^12^ Minato *et al.* experimentally verified this prediction and reported larger force and displacement outputs for actuators with locally thicker PPy at the tip and base ends, at the expense of a loss in the actuator speed.^13^ Gaihre *et al.* fabricated trilayer actuators with 110 μm and 32 μm-thick Au-coated PVDF electrolytes and 8 μm-thick electropolymerised PPy electrodes, and reported larger but slower displacement for the thinner actuator.^14^ The cause of the slower response was that thinner layers of Au were sputtered for the thinner actuator to avoid short circuiting, which led to lower conductance. Khalili *et al.* developed two mathematical models for the multi-objective optimisation of conductive polymer-based trilayer actuators.^15,16^ These models showed that thicker PPy electrodes resulted in smaller tip displacements and larger blocking forces. Põldsalu *et al.* fabricated trilayer actuators through inkjet printing of 1.9, 3.3, 7.3 μm-thick poly(3,4-ethylenedioxythiophene)polystyrene sulfonate (PEDOT:PSS) electrodes and 3.1, 5.2, 10.5 μm thick PEDOT:PSS/activated carbon aerogel electrodes on 125 μm-thick porous PVDF electrolytes.^17^ They reported larger strain from the actuators with thicker electrodes due to larger force generated to overcome the stiffness of the much thicker PVDF electrolyte layer.

The development of microfabrication strategies has enabled the creation and investigation of ultrathin (<20 μm) trilayer microactuators.^18–23^ Maziz *et al.* fabricated microactuators with spin-coated poly(ethylene oxide)/nitrile butadiene rubber (PEO/NBR) interpenetrating polymer networks and vapour-phase-polymerised poly(3,4ethylenedioxythiophene) (PEDOT) electrodes, which were reported to have a fast response and kHz frequency actuation amplified by mechanical resonance.^19^ Among the actuators with 6, 12, and 19 μm-thick electrolytes, the thinnest actuator showed the lowest PEDOT strain, which was explained by the more pronounced short circuiting through the electrolyte in the thinner samples.^19^ By applying a transmission line model, Takalloo *et al.* demonstrated how changing different parameters could affect the performance of ultrathin trilayer microactuators.^24^ They showed that with a lower electrode thickness, the deflection angle is either unaffected or increased, the blocking force decreases, and the actuation speed increases; with a lower electrolyte thickness, the deflection angle increases, the blocking force decreases, and the actuation speed increases. However, experimental results showing these trends have been inadequate.

A quantitative understanding of the thickness dependence backed by statistically reliable agreement between experiments and modelling is crucial for the rational design of ultrathin microactuators and accurate prediction of actuation behaviour. The aforementioned studies have shown that experiments on trilayer actuators, especially ultrathin ones, can be affected by confounding variables such as short circuiting, variations in contact resistance, asymmetry, and non-uniformity, which may cause discrepancies between experimental results and theoretical predictions. Such confounding variables should either be eliminated during the fabrication procedure or taken into account in the models.

Our previous work introduced a novel technique that made use of a microscale additive manufacturing method, namely aerosol jet printing (AJP), to fabricate trilayer actuators in their entirety.^23^ This technique enables low-cost rapid prototyping and micropatterning of ultrathin actuators with lateral resolution down to 10 μm. In addition to the potential for creating sophisticated micropatterned actuator prototypes, the technique facilitates convenient fabrication of actuators with varying dimensions and compositions, and thus is suitable for investigating the dependence of actuation performance on different factors such as electrolyte and electrode thicknesses.

In this paper, we have used AJP to construct ultrathin high aspect-ratio electrochemical microactuators that consist of Nafion electrolyte layers (with hydrated H^+^ ions) and PEDOT:PSS electrodes. Similar to our previous work,^23^ the bending of the actuators is driven by ion insertion and removal at the electrodes with fast reduction and oxidation of PEDOT. Here, integrated Au contacts and thin Nafion encapsulation layers are employed to overcome issues of loose electrical connection, short circuits, and delamination. The actuating cantilevers are 3.1 mm long and 0.4 mm wide, with 3 × 0.2 mm^2^ electrodes. By changing the number of printing passes, the actuators are fabricated with varying layer thicknesses (0.36 μm to 1.9 μm-thick electrodes, and 3.5 μm to 12 μm-thick electrolytes). These actuators are then tested under DC voltages from 0.2 V to 0.8 V. The effects of electrode and electrolyte thicknesses on the amount of charge transfer, angular deflection, and actuation speed are then analysed and discussed.

## Experimental

### Fabrication

The actuators were fully aerosol-jet printed with an Optomec Aerosol Jet 200 Printer. Nafion ink was prepared by diluting Nafion™ perfluorinated resin solution (5 wt% in a mixture of lower aliphatic alcohols and water, Sigma-Aldrich, rebranded as Merck) in a 1 : 3 volume ratio with deionised water. The PEDOT:PSS ink used was Clevios™ PH 1000 PEDOT:PSS (1 : 2.5 w/w, 1.0–1.3 wt% in water, Heraeus). The Au ink used was UTDAu25 TE gold nanoink (25% w/v solution in proprietary organic solvents, UT Dots, Inc.). Different from our previous paper,^23^ in this work we used a pneumatic atomiser instead of an ultrasonic atomiser to print Nafion, showing that both are viable options. Before printing, ∼15 mL of Nafion ink was transferred to the pneumatic atomiser vial. Ethanol (Merck) was added to the pneumatic atomiser bubbler to introduce ethanol vapour in the nitrogen carrier gas. In occasional cases of foaming in the vial, a few drops of ethanol were added to destabilise the foam. PEDOT:PSS and Au were printed using the ultrasonic atomiser, with ∼2 mL of ink in respective ultrasonic atomiser vials. When PEDOT:PSS was printed, the ultrasonic atomiser vial was filled with deionised (DI) water to introduce water vapour in the nitrogen carrier gas. A nozzle of 300 μm was used throughout, which gave printed line widths of ∼40 μm depending on the ink and printing parameters, due to the focusing effect of the nitrogen sheath gas. The printing patterns were designed with AutoCAD. To form films, the areas were covered with a serpentine fill method. The thickness of each layer could be controlled by modifying the raster line separation, the number of printing passes, or the ink flow rates. The detailed printing parameters for each ink can be found in Table S1 (ESI†). The aerosol delivery tube was cleaned occasionally after several printing passes with DI water and blow-dried with compressed air to flush out the condensates. After all the printing was complete, all movable components of the printer were disassembled and ultrasonicated with a Branson Ultrasonics Buffing Compound Concentrate in a Branson Bransonic® CPX 3800 Ultrasonic Bath for 15 minutes, before rinsing with DI water and blow drying with compressed air.

**Fig. 1:**
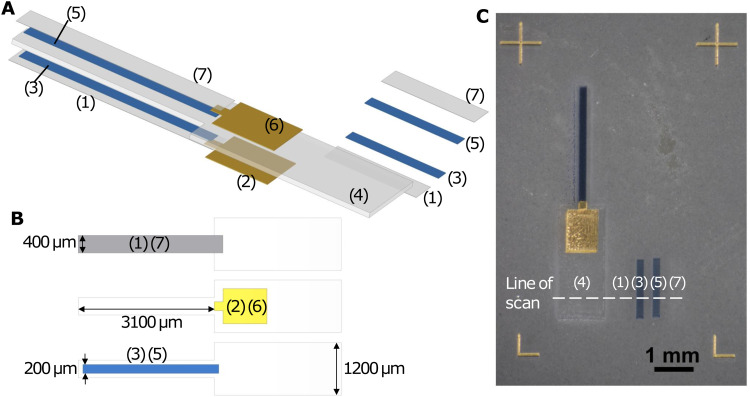
(A) Exploded diagram showing a multilayer structure of the AJP actuator and sequence of printing. The rectangular pieces on the right are thickness test samples. Layer 1: Nafion bottom encapsulation layer; layer 2: Au bottom layer; layer 3: PEDOT:PSS bottom layer; layer 4: Nafion electrolyte layer; layer 5: PEDOT:PSS top layer; layer 6: Au top layer; and layer 7: Nafion top encapsulation layer. The thickness of each layer is exaggerated ∼10 times in comparison to length and width. (B) Diagram showing the position of Nafion encapsulation layers (layers 1 and 7), Au layers (layers 2 and 6) and PEDOT:PSS layers (layers 3 and 5) relative to the Nafion electrolyte layer (layer 4). (C) Optical image of a printed actuator along with thickness test samples. The fiducial markers at the corners are printed to ensure accurate alignment of different layers. The thickness of layers 4, 1, 3, 5, and 7 are obtained by profilometer scanning along the direction shown with the dashed line.

In the printing session, Nafion, Au, PEDOT:PSS, Nafion, PEDOT:PSS, Au, and Nafion were sequentially deposited on a glass slide, forming a 7-layer structure as illustrated in the exploded diagram (Fig. 1A). The samples were cured in a Heratherm OGH 60 oven at 150 °C for 2 hours after printing. For release from the substrate, they were soaked in deionised water, peeled off from the glass slide with a pair of tweezers while being viewed under a stereomicroscope (KERN OZM 544), and then dried between paper towels.

As shown in Fig. 1A and B, the Nafion electrolyte layer (layer 4) was the thickest layer and it mechanically supported the other layers, ensuring that the actuator was freestanding and did not crumble. It had a narrow (400 μm) cantilever region for actuation and a wider (1.2 mm) base region for electrical connection to Kelvin clips and handling with tweezers. The PEDOT:PSS electrode layers (layers 3 and 5) were attached to the Nafion electrolyte layer. They were designed to be narrower (400 μm) than the electrolyte layer, leaving a 100 μm margin on both sides to prevent short-circuiting. The Au layers (layers 2 and 6) were attached to the PEDOT:PSS electrode layers nearer to the base of the cantilever and the Nafion electrolyte layer in this base region, serving as contact electrodes. They overlapped with the PEDOT:PSS layers by 100 μm in the lengthwise direction as ohmic contacts and were wider at the base region for clamping with Kelvin clips. This design helped bridge the narrow microactuator and the Kelvin clip connecting the device to external instruments while minimising the influence of clamping on the actuator performance.^25^ Fig. S1 (ESI†) shows alternative designs that did not incorporate Au layers. To clamp an actuator to a Kelvin clip with tweezers, the contact area on the actuator needed to be at least ∼1 mm wide for reliable handling and alignment by a human operator. It was also better to have large contact electrodes for firm electrical contact and low contact resistance. A wider PEDOT:PSS contact pad region at the base (Fig. S1A, ESI†), however, adds two ion reservoirs that participate in charging and discharging, and may add an exposed PEDOT:PSS contact pad area that participates in actuation. For rectangular actuators where the PEDOT:PSS width is the same at the contact pad and cantilever regions (Fig. S1B, ESI†), the design of narrower actuators is precluded and curling along the length of the actuator may occur for low-aspect-ratio actuators, reducing their ability to bend along the width.^13^ If the PEDOT:PSS electrode is kept rectangular and the Nafion layer is wider in the contact pad region to prevent short circuits (Fig. S1C, ESI†), narrower cantilevers can be made, but the contact electrode is small. In all designs without Au, the actuating length of the cantilever depends on the clamping position. As such, the incorporation of Au electrodes was found to reduce the number of confounding variables and made subsequent actuation testing more reliable. Layer 1 and layer 7 were thin Nafion encapsulation layers to prevent delamination of PEDOT:PSS from the Nafion electrolyte. As demonstrated in Fig. S2 (ESI†), without the encapsulation layers, thicker PEDOT:PSS layers tended to detach from Nafion and get left on the glass slide during peel-off. In each printing pass of the Nafion encapsulation layers and PEDOT:PSS layers, we also printed a rectangular sample to one side for subsequent profilometry measurements (Fig. 1A and C), providing a way to estimate the thickness of the corresponding layers in the actuator. We printed 6 actuators of different Nafion electrolyte thicknesses and 6 actuators of different PEDOT:PSS electrode thicknesses, by varying the number of printing passes when printing these layers.

### Characterisation and actuation tests

Optical images and videos of the actuators were taken with a KERN OZM 544 stereomicroscope on which a Canon EOS 2000D DSLR camera was mounted. Thicknesses of the Nafion and PEDOT:PSS layers were measured with a DektakXT profilometer (stylus radius: 2 μm; stylus force: 29.4 μN; scan length: 4000 μm; scan duration: 20 s). The mean thickness of each layer was obtained by averaging the plateau region height of four scans with different positions in the direction of the width of the actuator/rectangle (Fig. 1C). Cross-sectional scanning electron microscopy (SEM) images were taken with a TM3030 Plus Tabletop Microscope, with a 15 kV accelerating voltage in the backscattered electron mode. The cross-section of the microactuator with 4 PEDOT:PSS printing passes was obtained by cutting with a razor blade, after the actuation tests.

The DC actuation tests followed procedures described in our previous work.^23^ The actuators were clamped at the Au contact electrodes with a Kelvin clip that consisted of a 3D printed holder on which copper tape was affixed for electrical connections. Voltages of 0.2 V, 0.4 V, 0.6 V, and 0.8 V were applied with an IT6412 bipolar DC power supply connected to a Devantech USB-RLY08C relay board, which was used to power and electrically short the actuator successively in intervals of 50 s. The voltage across the actuator was measured using a Keithley 2100 digital multimeter. Current measurements were obtained by measuring the voltage across a 330 Ω series resistor with another digital multimeter. The video recording, relay switching, and recording of measurements were all controlled using a LabVIEW (version 21.0.1) programme. Motion tracking was carried out in a blender (version 3.2.0), and the coordinates of track points were exported with a script on the Blender Python API, enabling bending angle calculations. For each actuator, the DC actuation test was repeated at least 6 times with both forward (0.2 V, 0.4 V, 0.6 V, and 0.8 V) and backward (−0.2 V, −0.4 V, −0.6 V, and −0.8 V) voltages. Durability tests and cyclic voltammetry (CV) were conducted by replacing the DC power supply with an RS Pro RSDG830 function generator.

## Results and discussion

**Fig. 2:**
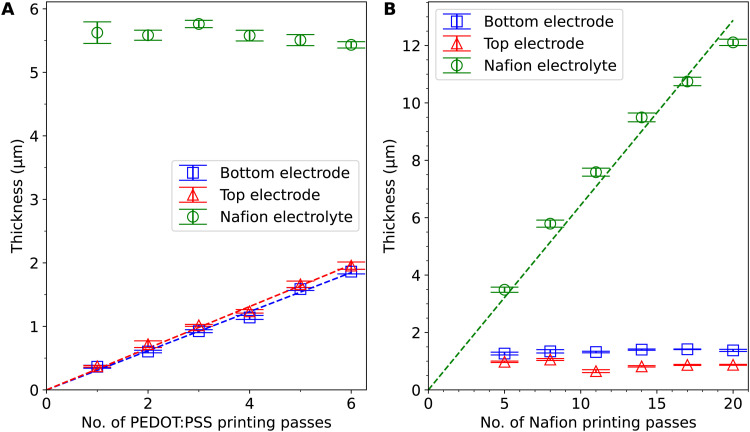
Thicknesses of PEDOT:PSS and Nafion against the number of printing passes in (A) 6 actuator samples with PEDOT:PSS thickness variation and (B) 6 actuator samples with Nafion thickness variation. The error bars indicate the standard deviation of results from 4 profilometry scans with Bessel's correction. The dashed lines are linear fits of the data points through the origin.

Fig. 2 shows the thicknesses of the PEDOT:PSS electrode layers and the Nafion electrolyte layers in the 12 actuators printed. The 6 samples in Fig. 2A have varied PEDOT:PSS thicknesses and the 6 samples in Fig. 2B have varied Nafion thicknesses. The thickness measurements show a linear trend against the number of printing passes. The deviation from a linear fit and the difference between the top and bottom layers are possibly due to the decrease in volume and the change in the concentration of ink in the vials, temperature drift and a change in aerosol delivery tube cleanliness over time in the printing process. The printing consistency can be further improved with more frequent clearing of condensates in the tube, more thorough cleaning of the equipment parts, more frequent (or continuous) refilling and replacement of ink, and optimisation of the ink composition and printing parameters. The cross-sectional SEM images of the actuator with 4 PEDOT:PSS printing passes (Fig. S3, ESI†) show a total thickness of ∼7.6 μm. Compared to the profilometry results which give a total thickness of ∼8.6 μm, the thickness measured by SEM is slightly smaller, probably because of water loss under vacuum, compression at the cutting edge, and inaccuracies from sample tilt and drift. The curling of PEDOT:PSS at the cutting edge also makes it difficult to accurately measure the individual layer thicknesses. Since the profilometry method avoids these problems and is non-destructive, we opted to use it over SEM for thickness estimation.

The range of thicknesses that can be achieved is limited by the AJP process: it is difficult to achieve larger PEDOT:PSS thicknesses (>2 μm) because if the PEDOT:PSS layers are thicker, they can come off prematurely during printing as they solidify after a larger number of printing passes. It is also difficult to achieve smaller Nafion electrolyte thicknesses (<3 μm) because the Nafion electrolyte layer provides mechanical support for the actuator to be freestanding. If the Nafion electrolyte layer is thinner, the actuators can fold on themselves during the peel-off process and be difficult to handle with tweezers. The Nafion encapsulation layers have a smaller thickness (0.33 ± 0.07 μm) and smaller stiffness^23^ than the electrode layers and thus are likely to have little effect on actuation.

**Fig. 3:**
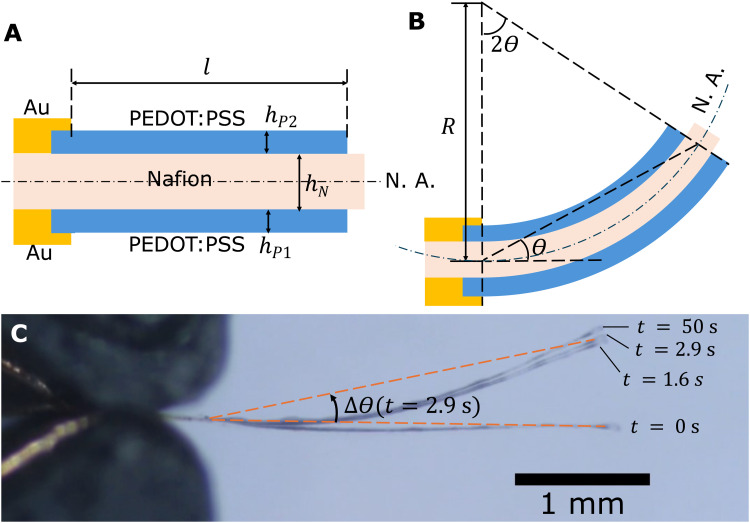
(A) Schematic showing the dimensional parameters for an AJP actuator in the neutral position before bending. The dashdotted line labelled ‘N. A.’ indicates the neutral axis. (B) Schematic of an AJP actuator under actuation, showing the relationship among the bending angle *θ*, actuator length *l*, and radius of curvature *R*. (C) Overlaid snapshots showing actuation of an actuator with 5.6 μm Nafion electrolyte thickness and 1.1 μm PEDOT:PSS thickness, at an applied DC voltage of 0.8 V. The actuator achieves 90% maximum charge transfer in 1.6 s and 90% maximum deflection in 2.9 s.

Side views of an actuator with Nafion electrolyte thickness *h*_N_ and PEDOT:PSS electrode thicknesses *h*_P1_ and *h*_P2_ are depicted in Fig. 3. It is observed that the overlap region between Au and PEDOT:PSS remains still when the actuator is actuated due to the stiffness of the Au layer and inadequate voltage for Au-based actuation (Fig. 3C). Thus, the active length that participates in actuation, *l*, is between the two endpoints of the cantilever region of the PEDOT:PSS electrode (indicated in Fig. 3A), which is 3000 μm in our design. The angular deflection *θ* is defined as the angle between the initial neutral axis and the line connecting the two ends of the active length. If we assume the actuator bends into an arc, by the alternate segment theorem, the angle subtended by the active part of the arc is twice the angular deflection. It follows from geometry that

1 2*θR* = *l*

where *R* is the radius of curvature.

The actuators sometimes have an initial bending angle *θ*_0_ before actuation. This is a result of the fabrication procedure which can cause asymmetry in top and bottom electrode thicknesses and Young's moduli. When analysing the recorded videos, we subtracted *θ*_0_ from the bending angle to obtain deflection Δ*θ* = *θ* − *θ*_0_. In our modelling, we assumed no pre-strain (*θ*_0_ = 0 and Δ*θ* = *θ*) for simplicity.

The voltage, current, charge, and deflection are plotted against time for the DC actuation tests (Fig. S4 and S5, ESI†). We can observe slight creeps, but no back relaxation, from the deflection-time graphs. We used two-term exponentials to fit the current and deflection data to obtain the transferred charge (Δ*Q*) and angular deflection (Δ*θ*) at steady state, as described in our previous paper.^23^ It is evident from Fig. S6 and S7 (ESI†) that both Δ*Q* and Δ*θ* at steady state are proportional to the applied voltage (*V*_appl_).

Protons and holes are the charge carriers in the actuator. Holes are transported in the network of PEDOT chains in the electrode and between PEDOT and Au contacts. Protons are transported in both the electrodes and the electrolyte, forming electrostatically attracted pairs with PSS^−^ anions, giving rise to volumetric capacitance.^26^ When the actuator is biased, PEDOT oxidation occurs at the anode with hole injection and proton deintercalation, whereas PEDOT reduction occurs at the cathode with hole extraction and proton intercalation. The charge transport in the actuator can be described by a transmission line model consisting of resistors and capacitors (Fig. S8, ESI†).^24,27–30^ In the steady state, the current in the circuit drops to zero and electric potential only drops across the capacitors. Assuming the uniform thicknesses and material properties of PEDOT:PSS along the length of the actuator, the circuit can be effectively treated as two capacitors with volumetric capacitance *C*_v_ connected in series representing the two electrodes. The capacitances of the two PEDOT:PSS electrodes can be expressed as

2a *C*_P1_ = *C*_v_*wlh*_P1_

2b *C*_P2_ = *C*_v_*wlh*_P2_

where *w* is the width of the PEDOT:PSS electrodes (200 μm). The applied DC voltage *V*_appl_ is divided between the two capacitors:3
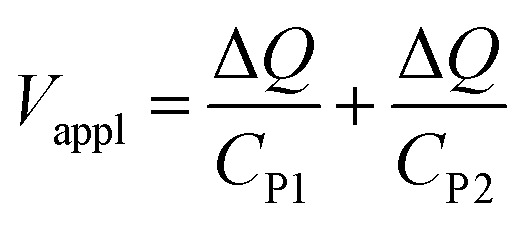


Substituting eqn (2a) and (2b) into (3), we get4
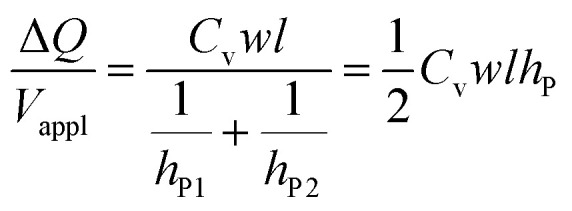
where *h*_P_ is the harmonic mean of *h*_P1_ and *h*_P2_.

**Fig. 4:**
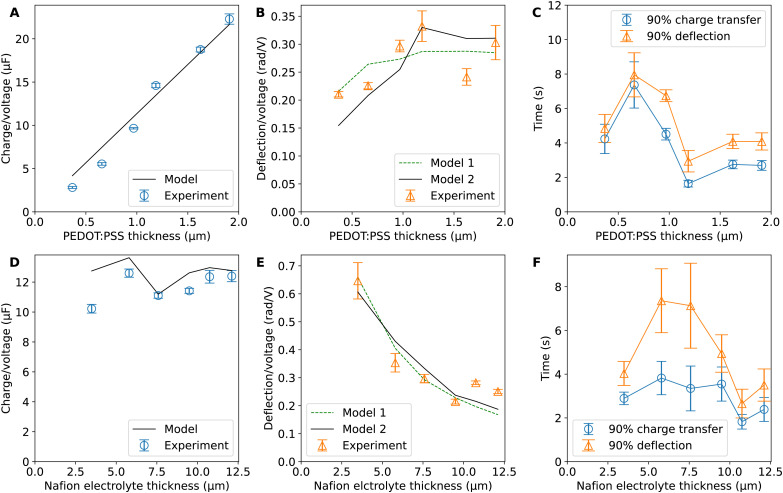
(A) Charge-to-voltage ratio 
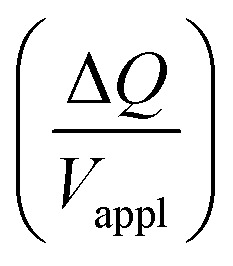
, (B) deflection-to-voltage ratio 
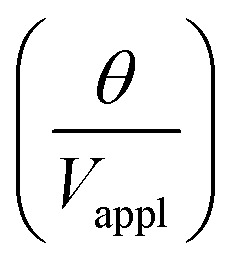
 and (C) time taken to reach 90% charging and 90% deflection for samples with different PEDOT:PSS electrode thicknesses (harmonic mean electrode thicknesses). (D) Charge-to-voltage ratio 
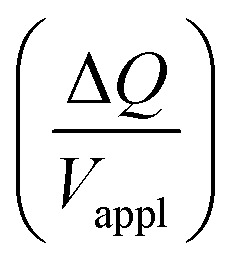
, (E) deflection-to-voltage ratio 
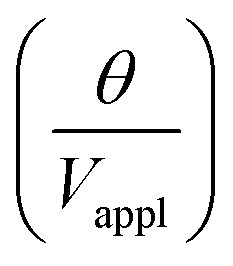
 and (F) time taken to reach 90% charging and 90% deflection for samples with different PEDOT:PSS electrode thicknesses. The modelling results in (A), (B), (D), and (E) are discrete points with the same *x*-values as the experiment data but are connected with lines for visualisation. The data points in (C) and (F) are also connected with lines for visualisation. Error bars for all plots indicate the standard deviation of data points with Bessel's correction. Note that while the models in (B) and (E) can be applied to asymmetric trilayers, which exhibit unequal forward and backward deflection-to-voltage ratios, the differences in our data are minimal as the actuators are largely symmetrical. Therefore, the forward and backward deflection data have been combined for each actuator due to the difficulty in consistently identifying which side is which.

Hence, the ratio of transferred charge to applied voltage at steady state, 
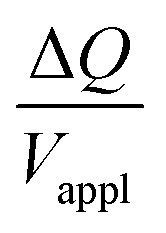
, is proportional to the harmonic mean of the electrode thicknesses. This proportionality is observed when 
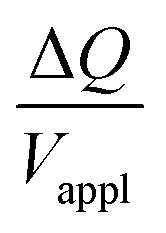
 is plotted against *h*_P_ (Fig. 4A). By fitting the experimental data using (5), we obtained *C*_v_ = 38 F cm^−3^, which is close to the value from the literature (39 F cm^−3^)^31^ and the value calculated from the slow scan cyclic voltammetry results (33 F cm^−3^) in our previous paper.^23^ We used the obtained *C*_v_ value and eqn (5) to predict 
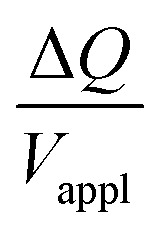
 for the samples with varying Nafion electrolyte thicknesses; the model is found to be in good agreement with the experimental data (Fig. 4D). The steady state transferred charge is not affected by the Nafion thickness, and the slight variations are due to the sample-to-sample variability in PEDOT:PSS electrode thicknesses from the fabrication process.

It has been proposed in the literature that in a conductive polymer actuator, the strain 
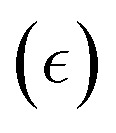
 in the conductive polymer is proportional to the charge density (*ρ*), with an empirical strain-to-charge ratio (*α*).^32^ Assuming a small strain (∼0.2%), the charge density can be approximated by dividing the charge transfer against the volume before actuation, and the strains on the two electrodes can be expressed as5a
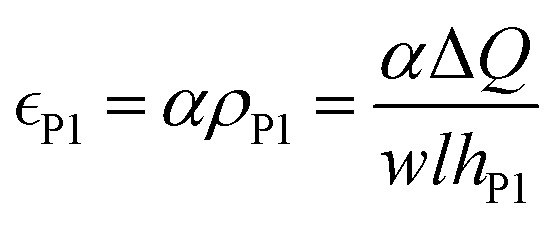
5b
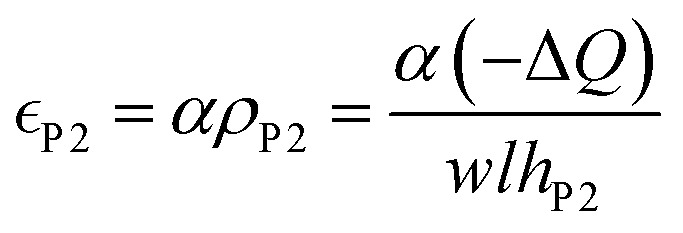
Substituting in eqn (4),6a
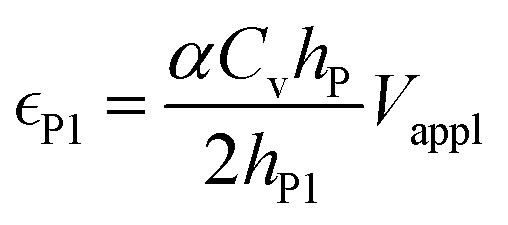
6b
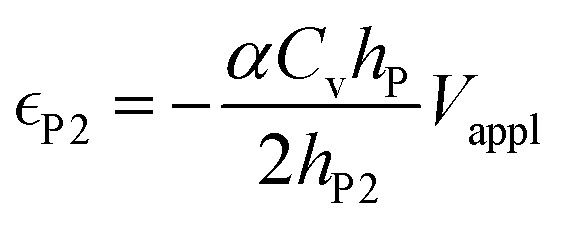


Bending is induced in the trilayer as a result of the strains in both electrodes. Following the multilayer bending model developed by Du *et al.*^33^ based on classical beam bending theory, the curvature of bending can be written as7
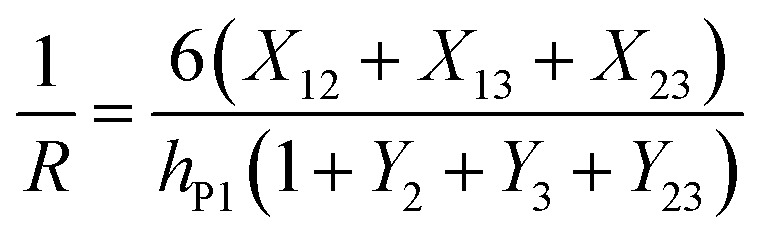
where

8a *X*_12_ = *m*_2_*n*(1 + *m*_2_)*ε*_P1_

8b *X*_13_ = *m*_3_(1 + 2*m*_2_ + *m*_3_)(*ε*_P1_ − *ε*_P2_)

8c *X*_23_ = *m*_2_*nm*_3_(*m*_2_ + *m*_3_)(−*ε*_P2_)

8d *Y*_2_ = 4*m*_2_*n* + 6*m*_2_^2^*n* + 4*m*_2_^3^*n* + *m*_2_^4^*n*^2^

8e *Y*_3_ = 4*m*_3_ + 6*m*_3_^2^ + 4*m*_3_^3^ + *m*_3_^4^

8f *Y*_23_ = *m*_2_*m*_3_[(4*m*_2_^2^ + 6*m*_2_*m*_3_ + 4*m*_3_^2^)*n* + 12(1 + *m*_2_ + *m*_3_)]

8g
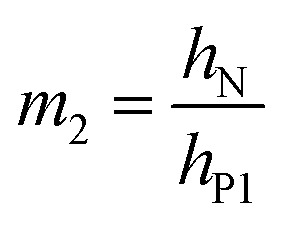
8h
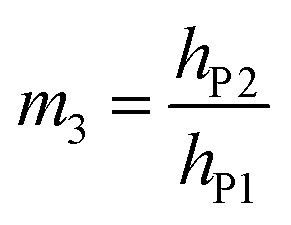
8i
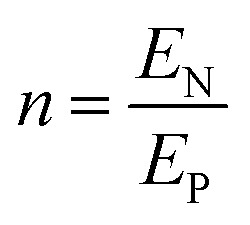
Here *E*_N_ and *E*_P_ are the Young's moduli of Nafion and PEDOT:PSS, respectively. Their values have been reported to exhibit non-homogeneity across the thickness after fabrication^34^ and *E*_P_ changes with the oxidation state during actuation.^6^ For simplicity, we treated them as constants, using values obtained by nanoindentation from our previous paper,^23^*E*_N_ = 2.5 GPa and *E*_P_ = 7.0 GPa.

**Fig. 5:**
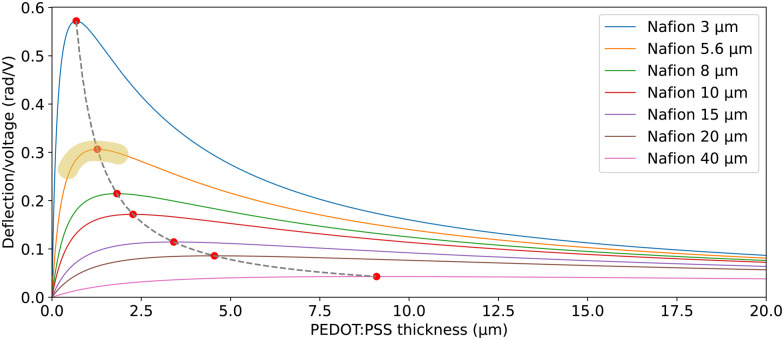
Predicted deflection-to-voltage ratio against PEDOT:PSS electrode thickness, for different Nafion electrolyte thicknesses. The dashed line traces the peak of the curves. The highlighted region shows approximately the range where the actuation tests of actuators with different PEDOT:PSS thicknesses (Fig. 4B) were conducted.

The deflection-to-voltage ratio, 
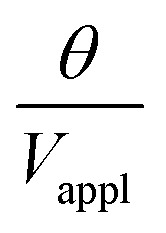
, can be predicted by substituting eqn (1), (8a)–(8i), and either (5a) and (5b) or (6a) and (6b) into eqn (7). With (6a) and (6b), 
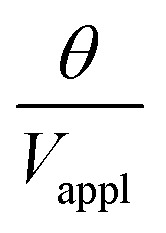
 is directly predicted from the dimensions of the actuator (model 1 in Fig. 4B and E). With (5a) and (5b), the experimental results of 
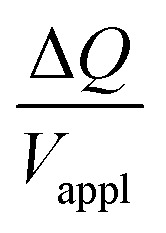
 are used (model 2 in Fig. 4B and E), which seem to give better fitting, possibly because of the inclusion of effects not captured by the constant *C*_v_ model. The results show that 
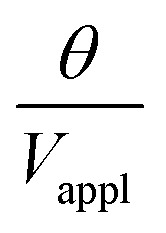
 has a large negative correlation with the Nafion electrolyte thickness and is less affected by the PEDOT:PSS electrode thickness for the range of dimensions investigated. It becomes clearer if we consider a symmetric trilayer (*h*_P1_ = *h*_P2_ = *h*_P_, 
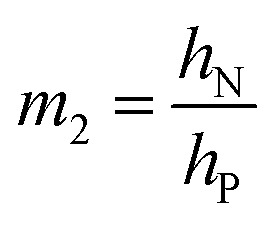
, and *m*_3_ = 1), and eqn (7) can be simplified^24,35^ to give9
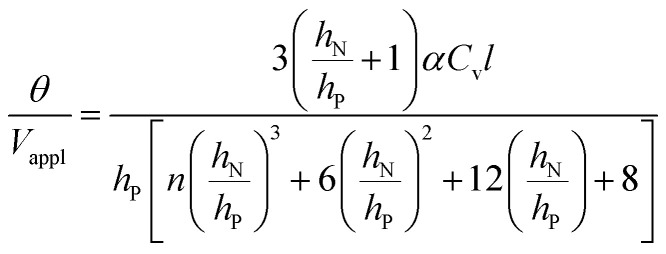
When 
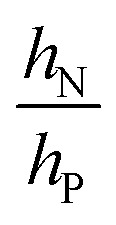
 is large and *n* is very small, 
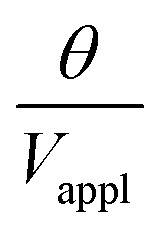
 depends predominantly on *h*_N_ and not *h*_P_. Using eqn (9), 
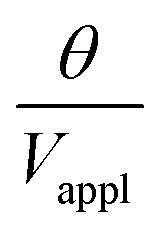
 can be plotted against the PEDOT:PSS thickness *h*_P_ by setting Nafion thickness *h*_N_ to different values (Fig. 5). With an increase in PEDOT:PSS thickness, the deflection-to-voltage ratio increases to a certain point before gradually decreasing. The experiments involving actuators with a Nafion electrolyte thickness of ∼5.6 μm and different PEDOT:PSS thicknesses (Fig. 4B) approximately correspond to the highlighted region in Fig. 5. With *h*_N_ = 5.6 μm, the optimal PEDOT:PSS thickness for the maximum deflection-to-voltage ratio is numerically determined to be ∼1.3 μm, to which the actuator with 4 PEDOT:PSS printing passes has the closest PEDOT:PSS thickness values (∼1.1 μm). In our experiments, we increased the PEDOT:PSS thicknesses up to ∼2 μm. Further increasing the PEDOT:PSS thickness likely results in gradually decreasing deflections. From Fig. 5, it can also be observed that the optimal electrode thickness increases with higher Nafion electrolyte thicknesses. More experiments can be performed in the future to confirm these predictions.

The peak and gradual levelling off effect of increasing the electrode thickness on deflection arises from a mechanism different from that of the IPMC examples mentioned in the Introduction.^9,10^ In IPMCs, charges are stored in the electrolyte and accumulate near the electrode–electrolyte interface. Initially, increasing the electrode thickness enhances the coverage of the electrode on the electrolyte, thereby increasing the charge storage capacity and charge density at the interface. However, once full coverage is achieved, a further increase in the electrode thickness has a minimal impact on charge storage, apart from increasing the conductance which may improve the actuation speed. Additionally, thicker metal electrodes increase stiffness, which reduces actuation. In contrast, for conductive polymer trilayers such as our actuators, charges are stored in the electrodes through intercalation and de-intercalation processes. The electrodes act as volumetric capacitors, meaning that increasing the electrode thickness proportionally increases charge storage capacity. However, the induced strain, which is proportional to charge density, is not affected by the increasing electrode thickness if the two electrodes have equal thicknesses (eqn (6a) and (6b)). With classical bending beam theory, we then predicted the peak and gradual reduction in deflection with an increase in PEDOT:PSS electrode thickness.

The strain-to-charge ratios, *α*, empirically obtained from fitting the PEDOT:PSS electrode and Nafion electrolyte thickness variation tests (model 2) are 0.44 × 10^−10^ m^3^ C^−1^ and 0.69 × 10^−10^ m^3^ C^−1^, respectively. The difference in these two values may be due to the voltage dependence of the strain-to-charge ratio, deviations introduced by the initial bending angle of the actuator and nonlinearities of the system, inaccuracies in the measurement of Young's moduli through nanoindentation on the rough AJP surfaces, and the effects of the encapsulation layers and the edge regions of Nafion. Considering isotropic expansion and contraction, the change in volume induced per unit charge can be deduced as 3*α*.^32^ The volume change per proton transferred can then be calculated, corresponding to a sphere of radius 0.17 to 0.2 nm. For comparison, the effective ionic radius of H_3_O^+^ can be approximated as the radius of a water molecule (0.138 nm),^36^ which corresponds to *α* ∼ 0.23 × 10^−10^ m^3^ C^−1^. This suggests that hydrated protons may be transported in the Eigen (H_9_O_4_^+^),^37^ Zundel (H_5_O_2_^+^),^38^ or other configurations with more hydration water molecules causing larger volume changes, or the transport of protons may be followed by osmosis afterwards (which likely plays a minor role in PEDOT:PSS as shown by Bonafè *et al.*^30,39^).

The model used in this study is a simplified representation of the actuation mechanism. It assumes that certain parameters, such as the strain-to-charge ratio, volumetric capacitance, and Young's modulus, remain constant throughout the process. In reality, these parameters can vary during actuation, particularly as the oxidation states of the electrodes change. Additionally, other factors such as ambient conditions, creep,^2,40^ nonlinear and time-dependent behaviours,^6^ property changes at the electrode–electrolyte interface,^41^ and more complex transport phenomena also need to be considered to give a more accurate description of the actuator's performance.

Fig. 4C and F show the average time to reach 90% of steady state charge transfer and deflection. It can be seen that the timescales for deflection follow the trends in timescales for charge transfer but are always slightly slower than charge transfer. This shows the close relation between charge transfer and actuation. This mechanical lag time can be caused by inertial effects, viscoelasticity, or osmosis after proton transport. The trends in timescales with respect to layer thicknesses are not apparent and datapoints have large spreads. This could be due to confounding variables such as contact resistance and ambient conditions. It may also be affected by creep due to prolonged stress in one direction, electrochemical processes during doping and de-doping, and the residual charge effect.^2,40^

Although the large spread of timescales makes it difficult to interpret this data, it is possible that thickness has little effect on the actuation speed for the dimensions investigated, because of the large length of the cantilever compared to its thickness. Ion transport across the electrode and electrolyte layers may not limit the speed as much as the electron (or hole) transport along the length of the actuator, which has a characteristic time constant^6,27^ given as follows:10
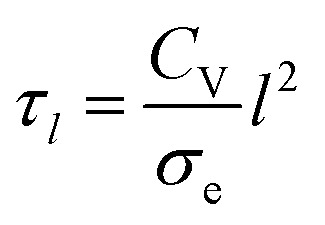
where *σ*_e_ is the electronic conductivity of PEDOT:PSS (0.5 S cm^−1^ according to our previous paper).^23^ The speed of hole transport along the actuator is not affected by thicker PEDOT:PSS because the reduction of resistance is offset by an increase in capacitance. *τ*_*l*_ was calculated here to be 6.8 s, which has the same order of magnitude as the results in Fig. 4C and F. The dominance of *τ*_*l*_ in the actuation timescale suggests that the actuation speed can be significantly improved by enhancing the electronic conductivity of the electrodes.

The prominent effect of length on the actuation speed is supported by preliminary tests on actuators with varying lengths (Fig. S9, ESI,† with thickness measurements shown in Fig. S10, ESI†). However, the data quality of these tests was impacted by factors such as creep, the large initial bending angle of longer actuators, the sharp current peaks and fast movements of shorter actuators, and the limited temporal resolution of the digital multimeter and camera, which made it difficult for motion tracking, curve fitting, and data interpretation. More repeats of the experiments will be performed in the future after further optimisation of the testing process. Besides, frequency response tests and time-dependent modelling will be performed to further elucidate the thickness effect on the actuation timescale.

To verify the robustness of the high-aspect-ratio design and explore the feasibility of higher voltages to improve actuation deflection, durability tests of an actuator were performed under 0.5 Hz sine waves with varying peak-to-peak voltages ranging from 0.4 V to 4.8 V (Fig. S11, ESI†). Minimal deterioration was observed after 360 cycles for peak-to-peak voltages up to 4.4 V. However, at a peak-to-peak voltage of 4.8 V, the actuator began to show instability and an irreversible drop in performance, possibly due to the onset of irreversible electrochemical reactions such as electrolysis of water. Fig. S12 (ESI†) shows CV at a scan rate of 50 mV s^−1^ conducted between ±0.8 V, ±1 V, and ±1.6 V. The CV curves are close to rectangular shapes for lower voltages, but more pronounced redox peaks and a drop in repeatability were observed for the ±1.6 V range, indicating a deviation from capacitor-like behaviours. Based on these findings and the possible residual charge effect at higher voltages,^40^ it is advisable to use low voltages for simple, linear, and cyclable actuation responses. The DC voltages used in this study are no more than 0.8 V, which is within the electrochemical stability window of the actuators.

The force that can be produced is another important metric of microactuators. According to the force model by Alici *et al.*,^42^ the blocking forces of our actuators are expected to be on the order of ∼μN, likely increasing with both PEDOT:PSS and Nafion thicknesses for the range of dimensions in this study. Measurements thus need to be carried out with force sensors having sensitivities in the ∼0.1 μN range, which will be addressed in future studies.

## Conclusions

We have built on the previous work on AJP electrochemical actuators to investigate the effects of electrode and electrolyte thicknesses on ultrathin high-aspect-ratio microactuators. Our unique fabrication and testing procedures enable precise fabrication and measurement of each layer with different thicknesses and easy mounting of microactuators on large low-impedance Au contact electrodes. It has been shown that increasing the electrode thickness linearly increases the amount of charge transferred and has a minimal impact on the angular deflection around and beyond the optimal thickness while increasing the electrolyte thickness has no effect on the charge transferred and reduces the angular deflection. Changing the thicknesses does not result in observable trends for actuation speed across the investigated dimensions, possibly because the actuation time scale is dominated by electronic conduction along the length of the PEDOT:PSS electrodes for these long and thin actuators. If maximising the deflection magnitude and speed is a goal, the Nafion electrolyte thickness should be reduced as much as possible, but it cannot be made too thin because it provides mechanical support to the whole structure in our current design. Nevertheless, it is possible to print new designs with other materials, such as polyurethane, to use as support at the base.^43^ The versatility of the AJP technique also enables the fabrication of actuators in other geometries, such as asymmetric actuators, kirigami-shaped actuators, and ones with abrupt or gradual thickness changes along the length.^44^ Additionally, actuators with other compositions and ion types could be studied. These efforts in exploring the parameter space will greatly enhance the understanding of microactuator behaviour and improve predictability in more complex systems. With further material and geometric refinements, the actuation performance will be significantly improved, making these high-aspect-ratio actuators suitable for applications such as biomedical devices that navigate tiny spaces within the human body and insect-scale robots for environmental monitoring.

## Data availability

Data for this article including profilometry data, AutoCAD files, electrical measurement and motion tracking data, codes to analyse the data, and videos are available at the Apollo repository at https://doi.org/10.17863/CAM.110646.

## Conflicts of interest

There are no conflicts of interest to declare.

## Supplementary Material

SM-020-D4SM00886C-s001
