# Supplementary material for: The thickness-dependent response of aerosol-jet-printed ultrathin high-aspect-ratio electrochemical microactuators
Source: Soft Matter. 2024 Nov 1;20(47):9424–33. doi: 10.1039/d4sm00886c (PMC11572699; doi:10.1039/d4sm00886c)
Supplement: SM-020-D4SM00886C-s001 [file SM-020-D4SM00886C-s001.pdf]

Supplementary Information (SI) for Soft Matter.
This journal is © The Royal Society of Chemistry 2024

# Supplementary Information

# Thickness-dependent response of aerosol-jet-printed ultrathin high-aspect-ratio electrochemical microactuators

Ji Zhang,[ab] Jeremy J. Baumberg*[b] and Sohini Kar-Narayan*[a]

[a] *Department of Materials Science & Metallurgy, University of Cambridge, 27 Charles Babbage Road, Cambridge CB3 0FS, UK. E-mail: sk568@cam.ac.uk*

[b] *NanoPhotonics Centre, Cavendish Laboratory, University of Cambridge, JJ Thomson Avenue, Cambridge CB3 0HE, UK. E-mail: jjb12@cam.ac.uk*

**Table S1. Printing parameters for each ink**

| | | **PEDOT:PSS** | **Nafion** | **Nafion encapsulation** | **Au** |
|---|---|---|---|---|---|
| Atomiser | | Ultrasonic | Pneumatic | Pneumatic | Ultrasonic |
| Atomiser current | | ~0.6 A | n/a | n/a | ~0.6 A |
| Bubbler | | DI water | Ethanol | Ethanol | DI water |
| Nozzle size | | 300 μm | | | |
| Ambient temperature | | ~20 °C | | | |
| Platen temperature | | 80 °C | | | |
| Chiller temperature | | 20 °C | n/a | n/a | 20 °C |
| Sheath flow rate | | 140 sccm | 140 sccm | 140 sccm | 140 sccm |
| Ink flow rate† | | 30-50 sccm | 520-550 sccm | 500-520 sccm | 15-23 sccm |
| Exhaust flow rate† | | n/a | 440-480 sccm | 450-460 sccm | n/a |
| Printing speed | | 5 mm/s | 5 mm/s | 5 mm/s | 5 mm/s |
| Raster line separation | | 20 μm | 20 μm | 40 μm | 20 μm |
| No. of printing passes | Varying PEDOT:PSS thickness | 1, 2, 3, 4, 5, 6 | 8 | 1 | 2 |
| | Varying Nafion thickness | 2 | 5, 8, 11, 14, 17, 20 | 1 | 2 |
| | Varying actuator length | 2 | 8 | 1 | 2 |
| Curing condition | | 150 °C, 2 hours | | | |

†The ink and exhaust flow rates are sometimes slightly adjusted to compensate for the change in appearance of aerosol deposition over time.

n/a: Not applicable; sccm: standard cubic centimetres per minute

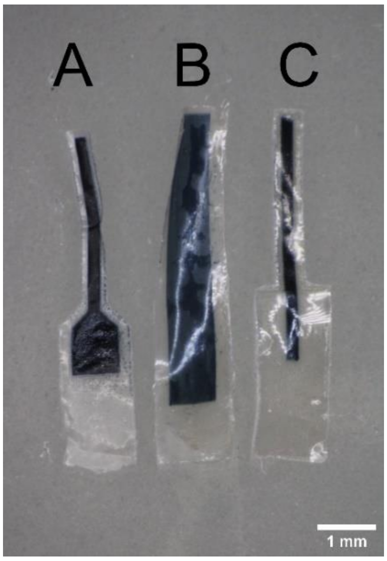


**Fig. S1.** Three PEDOT:PSS/Nafion/PEDOT:PSS actuator designs without incorporation of Au contact electrodes.

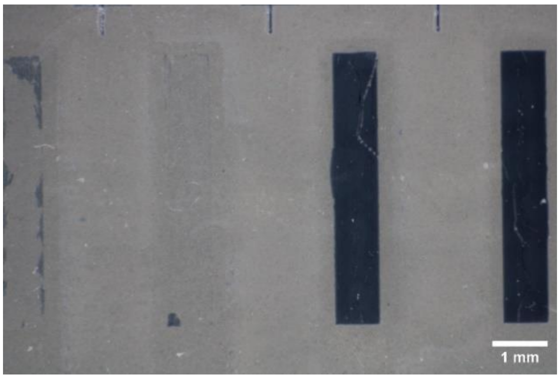


**Fig. S2.** Leftover bottom layer PEDOT:PSS after actuators (design A in **Fig. S1**) with different PEDOT:PSS thicknesses are peeled off. Left to right: 1 printing pass, 2 printing passes, 3 printing passes, and 4 printing passes.

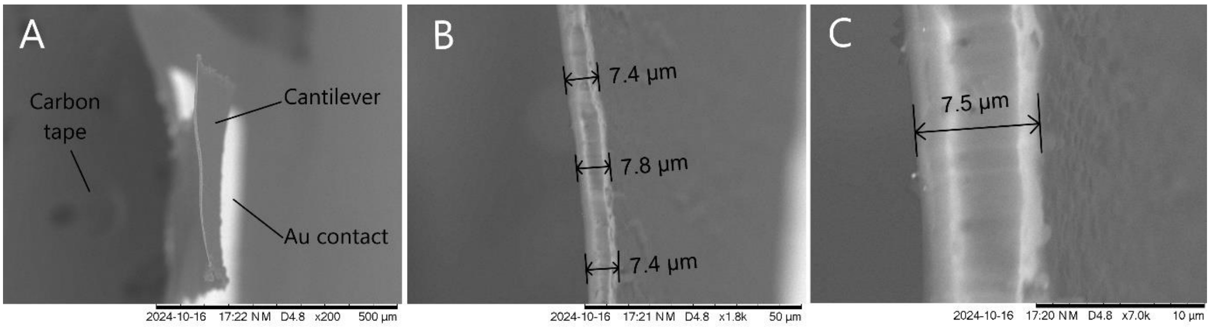


**Fig. S3.** SEM cross-sectional images of actuator with 4 PEDOT:PSS printing passes, taken at different magnifications.

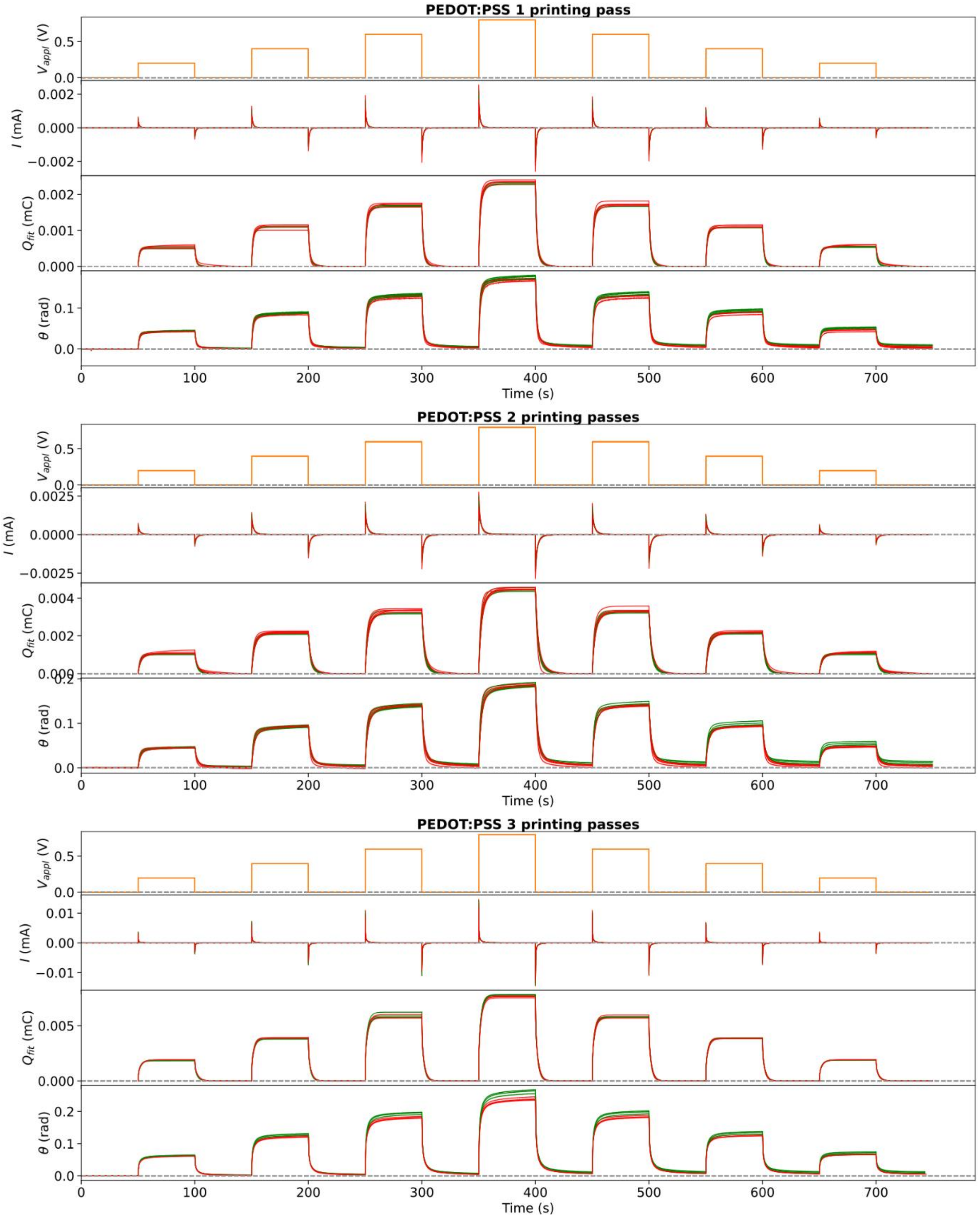


**Fig. S4.** Voltage, current, fitted charge, and deflection over time for DC actuation tests with varying PEDOT:PSS thicknesses. The red and green curves indicate voltage applied in both directions.

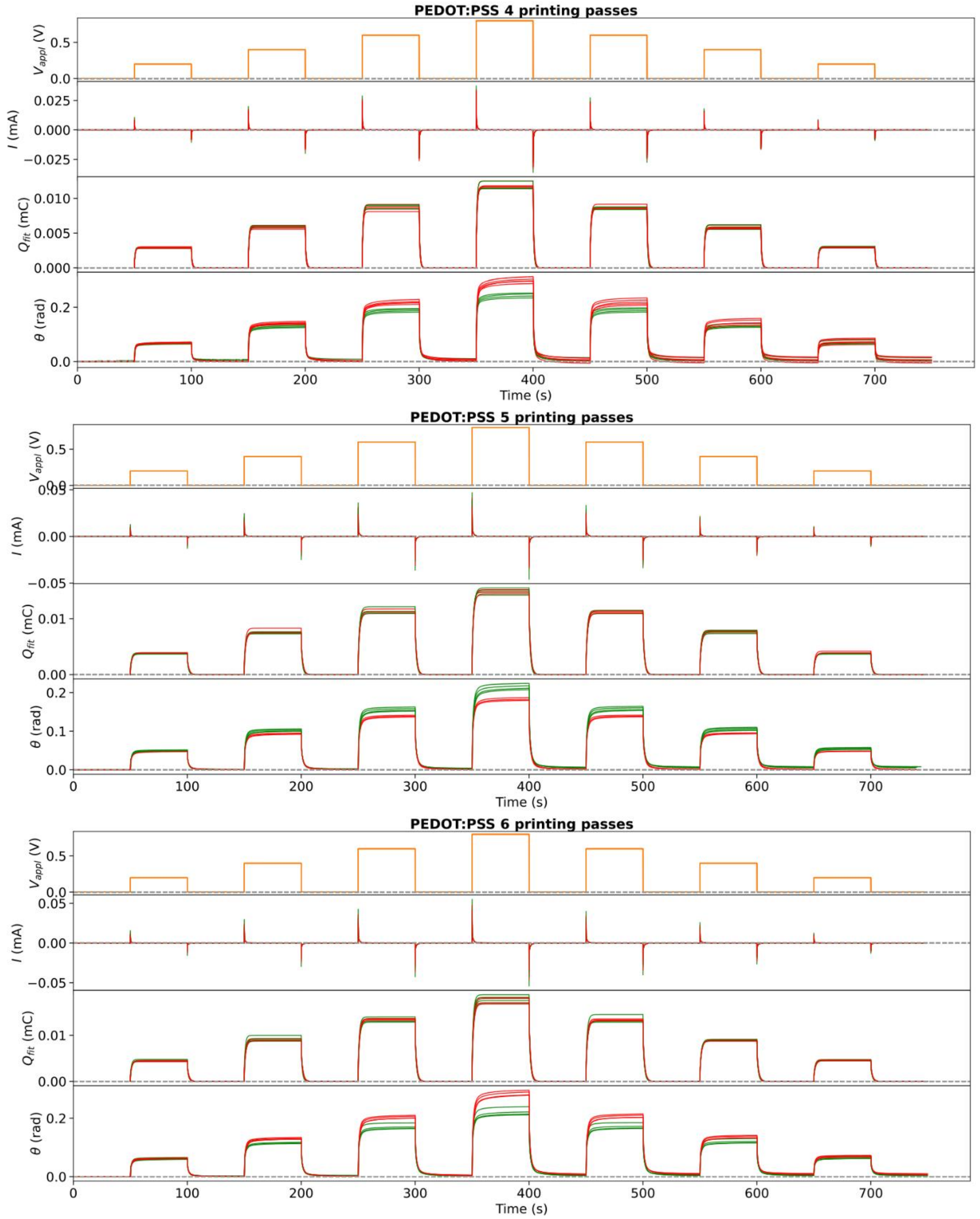


**Fig. S4 (cont'd).** Voltage, current, fitted charge, and deflection over time for DC actuation tests with varying PEDOT:PSS thicknesses. The red and green curves indicate voltage applied in both directions.

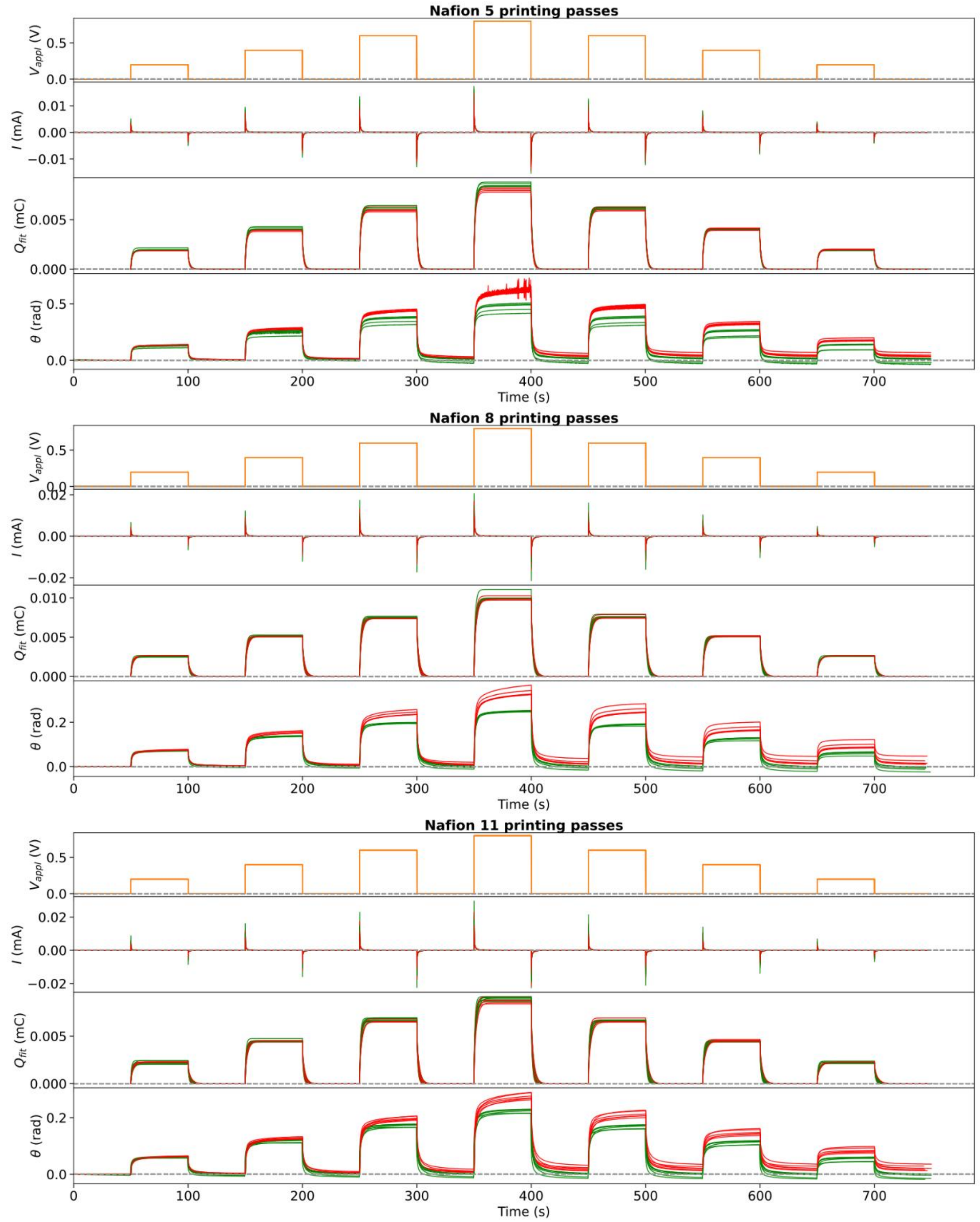


**Fig. S5.** Voltage, current, fitted charge, and deflection over time for DC actuation tests with varying Nafion electrolyte thicknesses. The red and green curves indicate voltage applied in both directions.

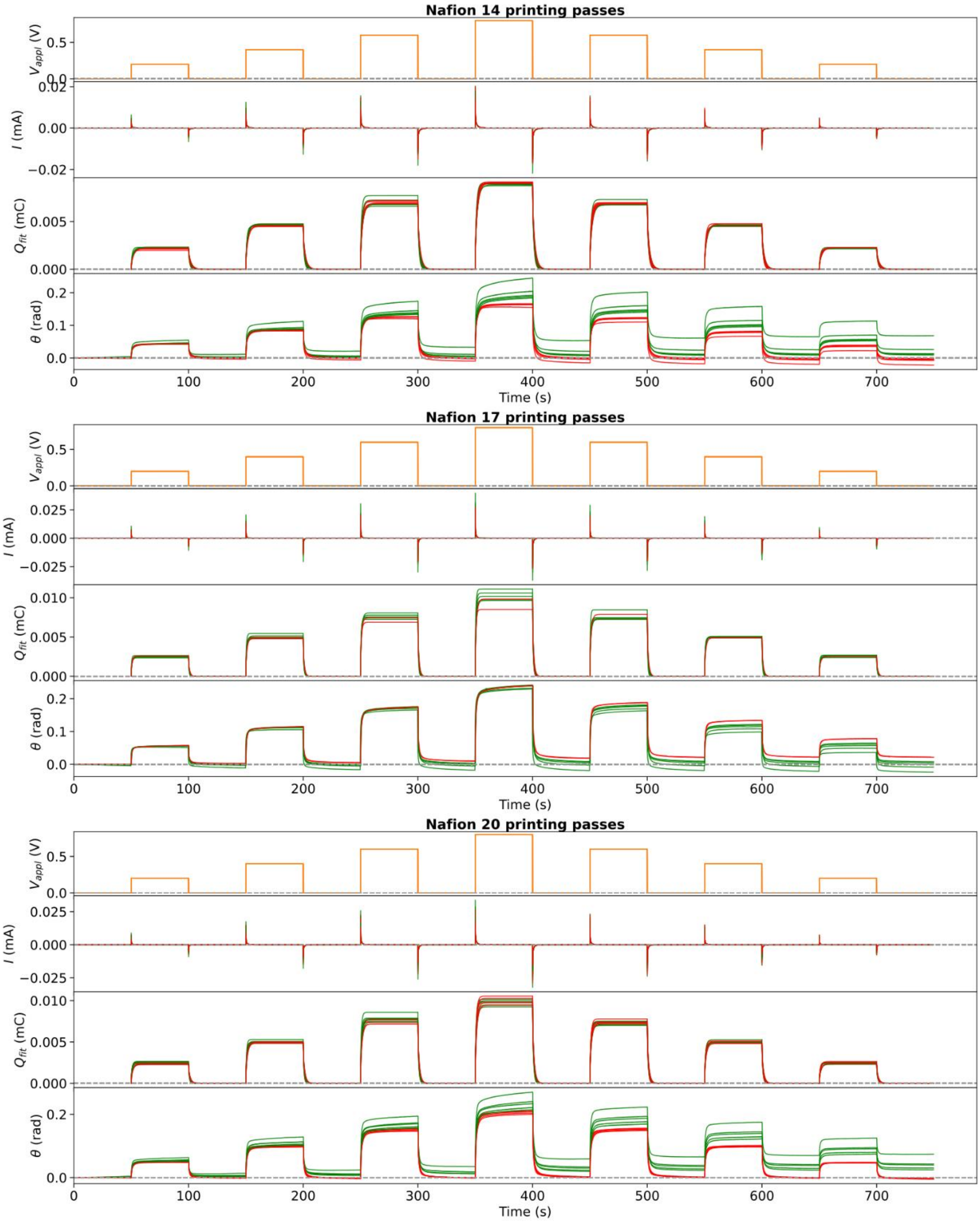


**Fig. S5 (cont'd).** Voltage, current, fitted charge, and deflection over time for DC actuation tests with varying Nafion electrolyte thicknesses. The red and green curves indicate voltage applied in both directions.

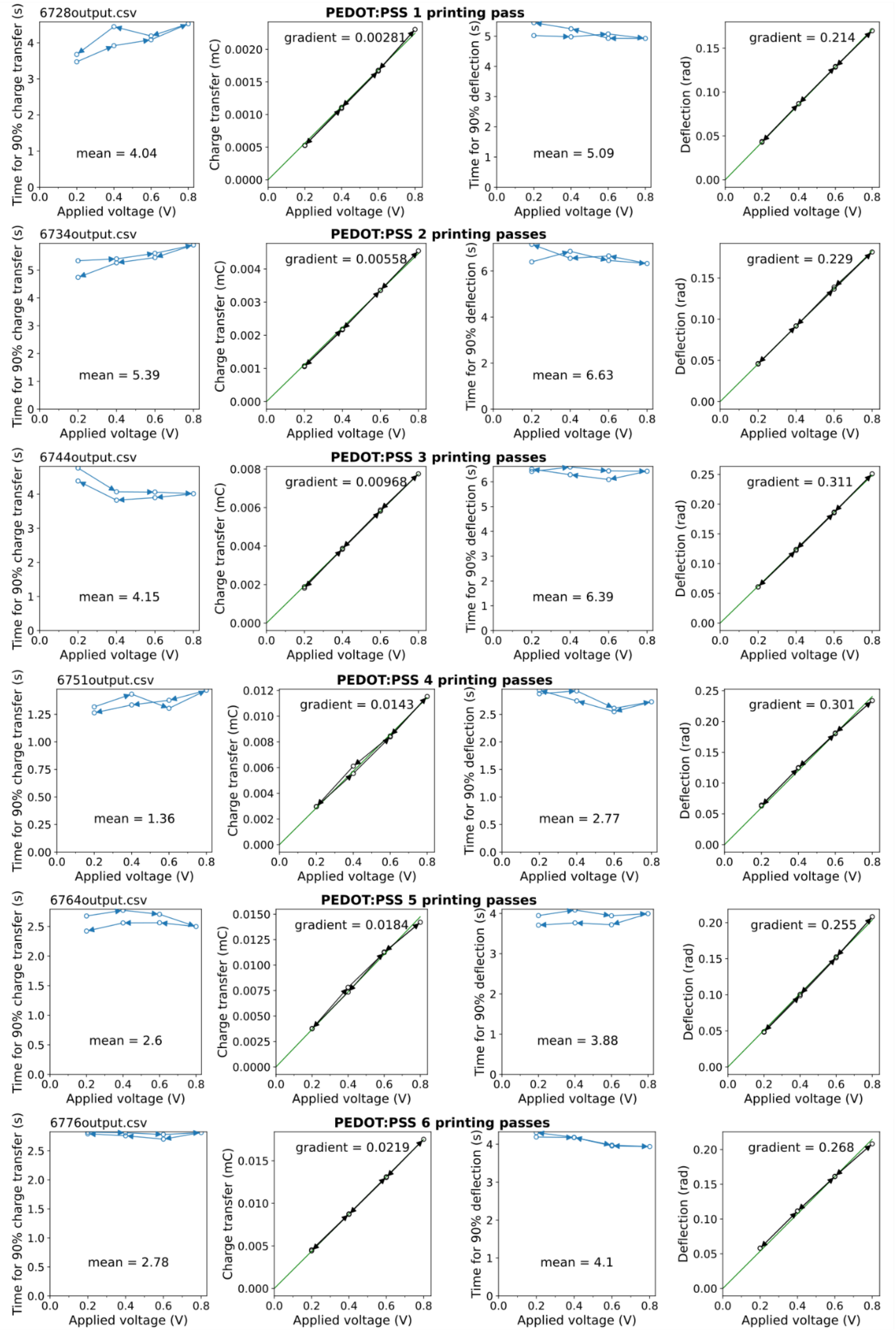


**Fig. S6.** Examples of actuation test analysis showing the dependence of steady-state charge transfer, steady-state deflection, and time to reach 90% of charge transfer/deflection on applied voltage for actuators with varying PEDOT:PSS thicknesses.

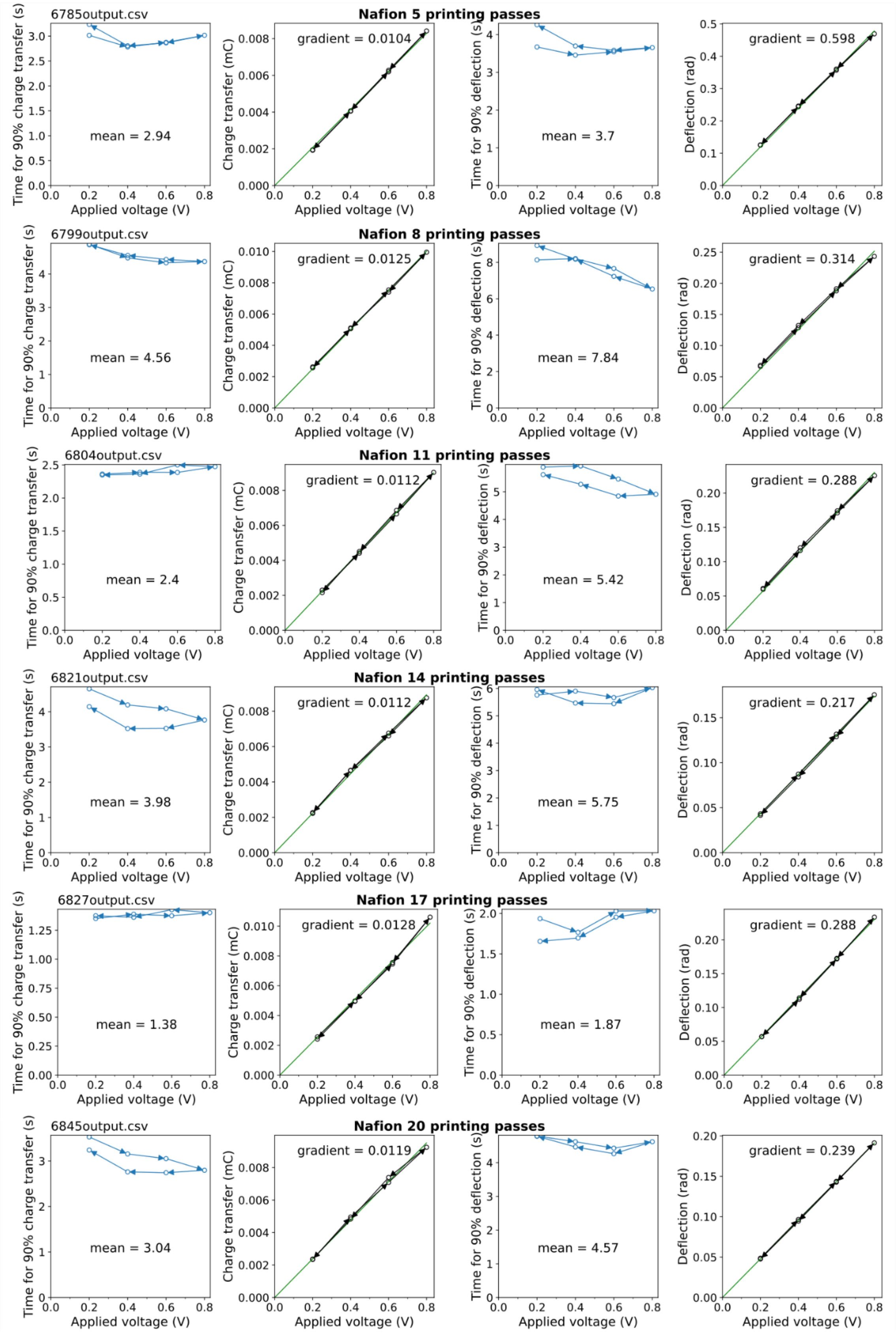


**Fig. S7.** Examples of actuation test analysis showing the dependence of steady-state charge transfer, steady-state deflection, and time to reach 90% of charge transfer/deflection on applied voltage for actuators with varying Nafion electrolyte thicknesses.

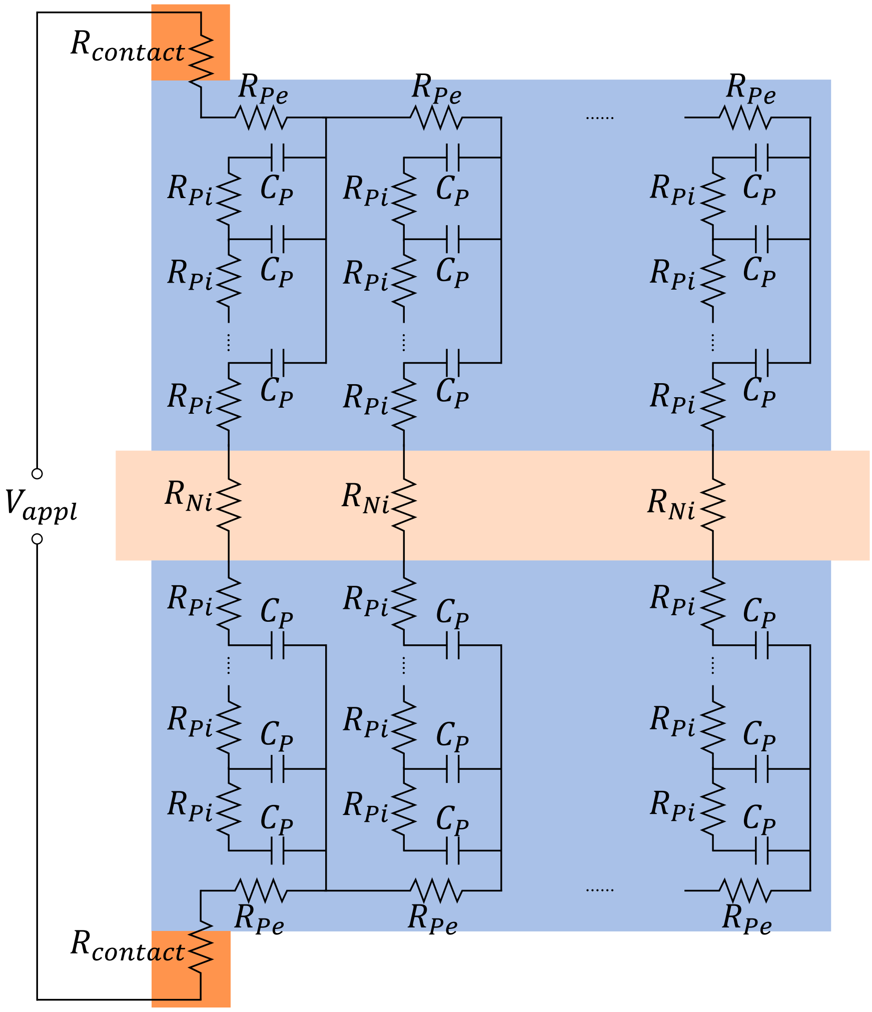


**Fig. S8.** Transmission line model of trilayer actuator.[1–5] The circuit elements $R_{Pi}$, $R_{Pe}$, and $C_P$ represent ionic resistance, electronic resistance and capacitance of small sections in PEDOT:PSS; $R_{Ni}$ represents ionic resistance across portions of the Nafion electrolyte; $R_{contact}$ represents the contact resistance. At steady state, the currents in all branches of the circuit are zero; thus, the electric potential only drops across the capacitors. Assuming uniform thicknesses and material properties of PEDOT:PSS along the length of the actuator, the potential in the Nafion electrolyte layer would be the same along the actuator. Hence, the capacitors in each PEDOT:PSS electrode can be combined and treated as one capacitor.

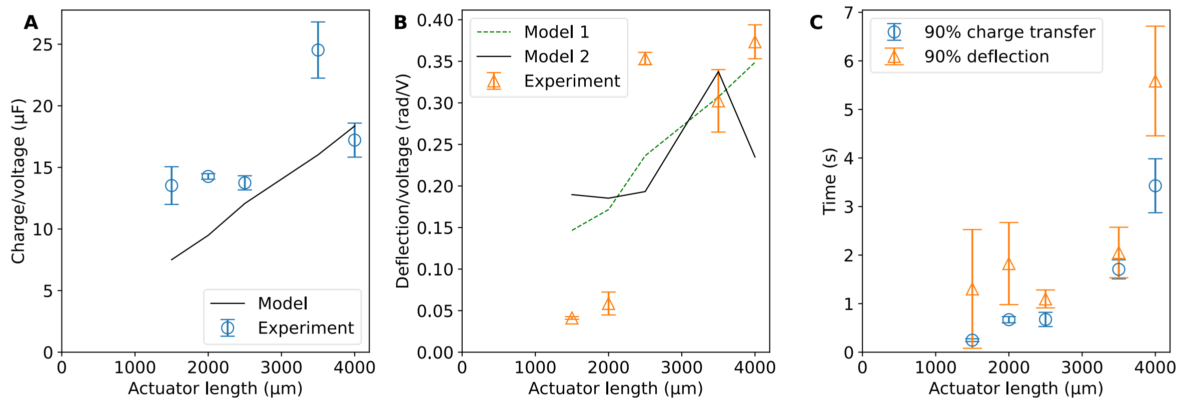


**Fig. S9.** **(A)** Charge-to-voltage ratio, **(B)** deflection-to-voltage ratio, and **(C)** time taken to reach 90% charging and 90% deflection for actuator samples with different lengths. The modelling results in (A) and (B) are discrete points with the same x-values as the experiment data but are connected with lines for visualisation. Error bars for all plots indicate standard deviation of data points with Bessel's correction.

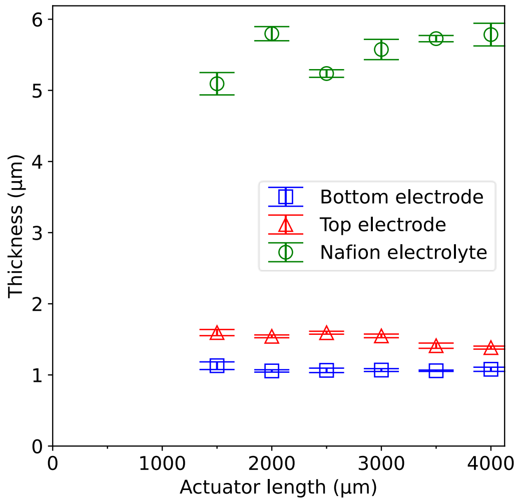


**Fig. S10.** Thicknesses of PEDOT:PSS and Nafion against number of printing passes in 6 actuator samples with length variation. The error bars indicate standard deviation of results from 4 profilometry scans with Bessel's correction.

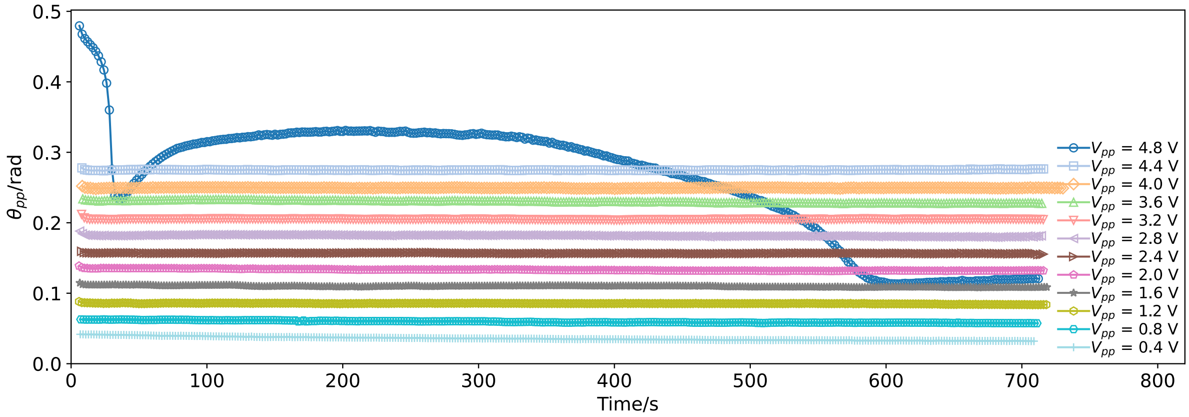


**Fig. S11.** Durability tests (of the actuator with 4 PEDOT:PSS printing passes) under sine waves at 0.5 Hz, for peak-to-peak voltages from 0.4 V to 4.8 V. Actuation showed minimal deterioration for more than 360 cycles for each voltage until the peak-to-peak voltage reached 4.8 V.

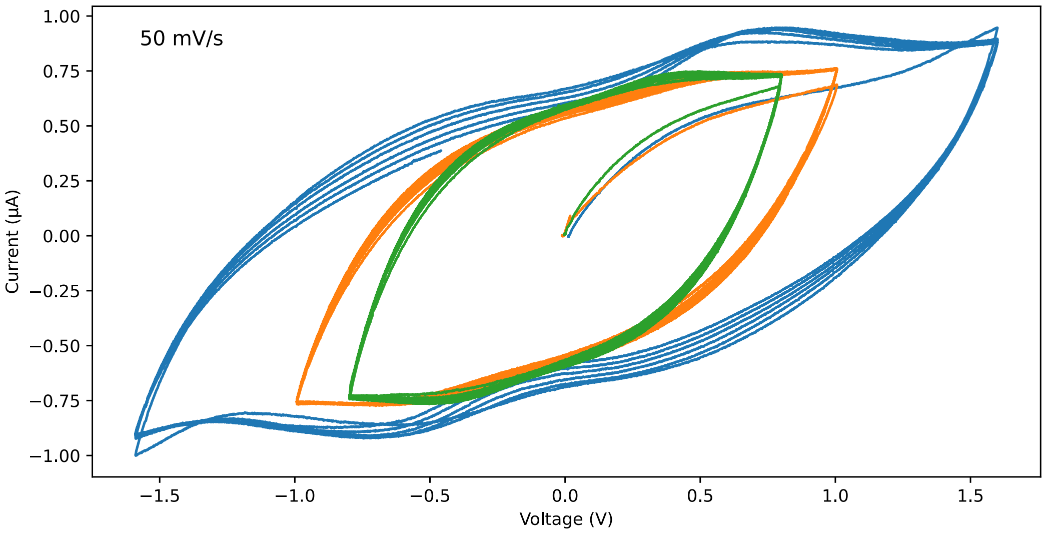


**Fig. S12.** Cyclic voltammograms (of the actuator with 4 PEDOT:PSS printing passes) measured at 50 mV s$^{-1}$ scan rate between ±0.8 V, ±1 V, and ±1.6 V.

# References


1 S. E. Takalloo, H. Seifi and J. D. W. Madden, in *Electroactive Polymer Actuators and Devices (EAPAD) 2017*, SPIE, 2017, vol. 10163, pp. 186–197.

2 T. Shoa, J. D. Madden, C. W. E. Fok and T. Mirfakhrai, *Advances in Science and Technology*, 2008, **61**, 26–33.

3 M. R. Warren and J. D. Madden, *Journal of Electroanalytical Chemistry*, 2006, **590**, 76–81.

4 T. Shoa, D. S. Yoo, K. Walus and J. D. W. Madden, *IEEE/ASME Transactions on Mechatronics*, 2011, **16**, 42–49.

5 F. Bonafè, C. Dong, G. G. Malliaras, T. Cramer and B. Fraboni, *ACS Appl. Mater. Interfaces*, 2024, **16**, 36727–36734.
